# In neuro critical care, capnia can be optimally controlled using a closed-loop ventilation system based on end-tidal CO_2_ signal (intellivent-asv^®^): preliminary results of a prospective interventional study

**DOI:** 10.1186/2197-425X-3-S1-A662

**Published:** 2015-10-01

**Authors:** L Piquilloud, A Polupan, I Matskovskiy, A Oshorov, D Novotni, T Laubscher, M Oddo, P Jolliet, JP Revelly

**Affiliations:** University Hospital of Lausanne, Adult Intensive Care and Burn Unit, Lausanne, Switzerland; Burdenko Neurosurgical Institute of Moscow, Intensive Care Department, Moscow, Russian Federation; Hamilton Medical Research, Bonaduz, Switzerland

## Introduction

Both hypo- and hypercapnia can be deleterious to brain injured patients. Due to the variability of CO_2_ production and elimination and to the unpredictable effects of ventilator settings changes, strict arterial CO_2_ partial pressure (PaCO_2_) control is difficult to obtain. Conceivably, using expired (end-tidal) CO_2_ as the input signal of closed-loop ventilation (Intellivent-ASV^®^) should optimize CO_2_ control compared to manual ventilator setting changes based on PaCO_2_ measurements.

## Objectives

The aim of this study was to compare PaCO_2_ evolution over time during standard controlled ventilation and during Intellivent-ASV^®^.

## Methods

Prospective interventional randomized study with a crossover design. Comparison of PaCO_2_ evolution during two sequential 2-hour periods of ventilation (standard ventilation and Intellivent-ASV^®^ version VUP02.11c in the “brain injury setting”), applied in random-order. A one-hour washout period was inserted between both periods. Arterial blood gas analysis was performed every 30 minutes. The number of manual settings adjustments made on the ventilator and actions performed to reduce intracranial pressure (ICP) were also recorded. Due to the small number of patients in this preliminary dataset, no statistical differences were tested.

## Results

**Figure 1:**
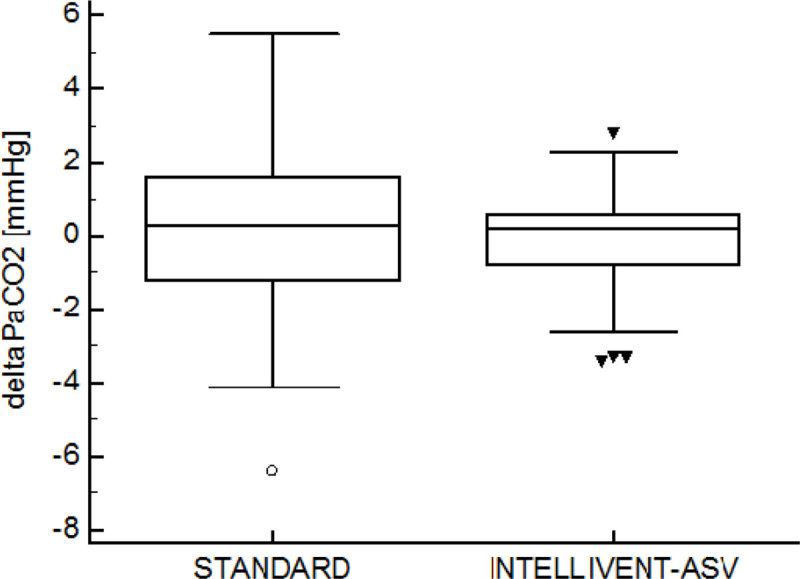
Figure 1

(medians [IQR]):11 patients were included (6 severe traumatic brain injury, 4 subarachnoid hemorrhage and 1 intra-cerebral hematoma). Age: 52 [42-54] years. Body mass index: 25.5 [24.7-27.0] kg/m^2^, GCS at admission: 6 [4-6.5]. SAPS 2 score: 42 [32-50]. PaCO_2_ was 36 [33-37] mmHg (range: 28 and 43 mmHg) during standard ventilation and 36 [34-37] (range: 30 and 43 mmHg) during Intellivent-ASV^®^. DeltaPaCO_2_ between two consecutive PaCO_2_ measurements are illustrated in Figure 1.

During standard ventilation, 22 settings adaptations were performed whereas only two manual adjustments were made during Intellivent-ASV^®^. Four (2 increase in sedation and 2 hypertonic saline administration) and 2 (increase in sedation) actions were performed in order to decrease ICP during standard ventilation and Intellivent-ASV^®^ respectively.

## Conclusions

The Intellivent-ASV^®^ CO_2_-regulated closed-loop ventilation mode can be safely used to deliver automated ventilation in brain injured patients. PaCO_2_ never reached extreme values and delta PaCO_2_ were very low during Intellivent-ASV^®^. Accordingly, fewer ventilator settings adaptations were required during Intellivent-ASV^®^. These preliminary results are promising and more patients must be included to evaluate the potential advantage of using Intellivent-ASV^®^ to optimize the control of capnia during neuro-resuscitation.

